# Prolapsus of the Vagina, Laceration of the Unimpregnated Uterus, and Protrusion of the Uterus

**Published:** 1847-03

**Authors:** 


*Prolapsus of the Vagina, Laceration of the Unimpregnated Uterus, and Protrusion of the Uterus.*—In the preceding page there has been recorded a case of laceration of the gravid uterus, with protrusion of the intestines through the rent, and the following case is an example of a similar injury in the unimpregnated condition. The patient was 60 years of age, and had borne seven children; she suffered under prolapsus of the vagina, and having never applied for medical advice, nor adopted any remedial measures, the affection proceeded from bad to worse; the sense of weight and dragging in the hypogastric region, the tenesmus, and dysuria became so severe, that she, by degrees, found herself less able to maintain the erect position, and was ultimately compelled to rest her head constantly upon her knees. On the 12th October, M. le Chaptois was called to her assistance; upon entering the room he was struck by the fetid cadaverous odour which exhaled from the bed in which the patient lay, in a state of profound prostration. An enormous mass of small and large intestines had protruded through the uterus, which was torn and dragged down by the mass; the womb was everted, and hung between the thighs like the finger of a glove; near the angle of the right Fallopian tube there was a laceration of nearly four inches in length, which had given passage to the intestinal mass. The patient had, for some hours, been speechless; the pulse was small and weak, and she was, to all appearance, moribund. M. le Chaptois, notwithstanding her desperate condition, having carefully washed and cleansed the viscera returned them with his hand into the pelvic cavity; they were then maintained in their place by the introduction of a sponge into the vagina. During the succeeding night the patient vomited frequently, and suffered from convulsions; but, after the bowels had been freely moved, an amelioration took place, and the case proceeded to a favourable termina ion.—*Ibid from Ibid.*

